# Modified Dolenc-Kawase anterior petrous rhomboid approach for petroclival meningioma: surgical nuances and complication avoidance

**DOI:** 10.3171/2022.1.FOCVID21256

**Published:** 2022-04-01

**Authors:** Ashish Suri, Ravi Sharma, Varidh Katiyar, Amol Raheja

**Affiliations:** Department of Neurosurgery, All India Institute of Medical Sciences, New Delhi, India

**Keywords:** petroclival, meningioma, cavernous sinus, Kawase’s triangle, rhomboid, anterior petrosectomy

## Abstract

Resection of petroclival meningiomas has remained challenging because of the critical neurovascular structures that lie in the vicinity, and thus various surgical corridors have been explored over time to figure out the optimum approach. In this video, the authors have highlighted the operative nuances of the modified Dolenc-Kawase (MDK) anterior petrous rhomboid approach. This approach gives access to the prepontine area, Dorello’s canal, anterior petrous apex, and upper two-thirds of the clivus with better angulation and surgical flexibility. It is a versatile approach for petroclival lesions that are not extending laterally and inferiorly to the internal auditory canal.

The video can be found here: https://stream.cadmore.media/r10.3171/2022.1.FOCVID21256

## Transcript

In this video, we attempt to highlight surgical nuances in a case of petroclival meningioma operated via modified Dolenc-Kawase (MDK) anterior petrous rhomboid approach.^1–3^ This was performed as a single-stage surgery spanning 10 hours.

### 0:34 Clinical Presentation.

A 48-year-old lady presented to us with previous failed attempt at tumor resection via retromastoid approach. On presentation, she had hoarseness of voice and swallowing difficulty since previous failed surgery. On examination, the gag was found to be impaired. There were no other cranial nerve or sensory motor deficits.

### 0:54 Preoperative Imaging.

On preoperative imaging, left petroclival lesion extending from the middle cranial fossa to the posterior cranial fossa was identified. It showed heterogeneous contrast enhancement and was isointense on T2-weighted magnetic resonance imaging (MRI). It had broad-based attachment to the left petrous ridge, clivus, and tentorium. Based on these findings, the likely radiological diagnosis was left petroclival meningioma.

### 1:22 Digital Subtraction Angiography (DSA).

DSA showed moderate cross-flow predominantly across the anterior communicating artery.

### 1:28 Decision-Making Regarding Surgical Approach.

An anterior petrosectomy approach was chosen in this case as the tumor was medial to the internal auditory canal (IAC), and this approach would provide an early control of the tumor vascularity and the cranial nerves would be encountered the last. Well-preserved hearing of the patient precludes addition of the posterior petrosectomy approach. While the previously failed retromastoid approach was avoided in view of expected adhesions, encountering cranial nerves early in exposure and the consequent need to work between the cranial nerves, and the inability to devascularize the tumor early via this approach.

### 2:05 Position.

Patient was positioned supine with 70°–80° head turn to the contralateral side with head in extension such that the zygomatic arch was at the highest point. The table is adjusted in beach-chair fashion with head-end elevation to decrease intracranial pressure and foot end elevated to increase venous return. The abdomen was prepared to harvest the fat graft for sealing the dural defect at the end of the surgery. Intraoperative neuromonitoring for trigeminal and facial nerves was also utilized.

### 2:40 Incision.

Left-side reverse question mark–shaped temporal scalp incision was given.

### 2:44 Craniotomy.

Left temporal craniotomy with zygomatic osteotomy was performed.

### 2:49 Temporal Base Drilling.

Temporal base was drilled to facilitate optimal access to lateral wall of cavernous sinus.

### 3:00 Deroofing of Superior Orbital Fissure.

Superior orbital fissure was deroofed using high-speed drilling to expose the V1.

### 3:10 Dissection of Posterior Cavernous Sinus.

These are a few images from our cadaveric demonstration to better understand the neuroanatomy of the desired region of interest, which shows the relationship between the various structures in the middle cranial fossa following interdural dissection of the lateral wall of the cavernous sinus.^4,5^

### 3:27 Division of Middle Meningeal Artery (MMA).

The foramen spinosum was widened and MMA was coagulated and divided.

### 3:41 Interdural Dissection of Cavernous Sinus.

Interdural dissection of cavernous sinus is a combination of blunt and sharp dissection. It is initiated in the region of V3 and then continued anteriorly over V2 and V1.

### 4:13 Sharp Dissection Over Greater Superficial Petrosal Nerve (GSPN).

The basal temporal dura is adherent to the endosteal layer in the region just behind V3. Sharp dissection over the GSPN avoids undue traction to the geniculate ganglion. Further dissection of the basal temporal dura from the gasserian ganglion facilitates exposure of the MDK anterior petrous rhomboid.

### 4:34 Middle Fossa Triangles and Drilling of MDK Anterior Petrous Rhomboid.

This is cadaveric image collage demonstrating the various triangles of the cavernous sinus and middle cranial fossa exposed during the MDK approach.^4,5^

### 4:55 Exposure of MDK Anterior Petrous Rhomboid.

The MDK anterior petrous rhomboid is bounded anteriorly by the proximal V3 and gasserian ganglion, laterally by the GSPN, posteriorly by the arcuate eminence, and medially by the petrous ridge.^3^

### 5:07 Drilling of MDK Anterior Petrous Rhomboid.

High-speed drilling was used to expose the posterior fossa dura by drilling the MDK anterior petrous rhomboid. Drilling was performed parallel to GSPN with the inferior extent of drilling till the inferior petrosal sinus and the posterior extent till the IAC.

### 5:27 Opening of Temporal and Posterior Fossa Dura and Tentorial Sectioning.

These cadaveric pictures demonstrate the stepwise dural opening technique and relationship between various neurovascular structures to the tentorium.

### 5:37 T-Shaped Basal Temporal Dural Incision.

Following the drilling, the basal temporal dura is opened in a T-shaped fashion.

### 5:58 Tentorial Sectioning.

Tentorial sectioning was done in multiple small incisions, carefully preserving the fourth cranial nerve in close proximity to the free margin of the tentorium.

### 6:17 Division of Superior Petrosal Sinus.

Superior petrosal sinus was coagulated and ligated to expose the tumor.

### 6:26 Tumor Debulking.

Tumor debulking is performed using an ultrasonic suction aspirator.

### 6:41 Use of Ultrasonic Aspirator.

It is essential to use the suction aspirator in a paintbrush fashion, and its motion should be parallel to the meningioma fibers.

### 6:48 Anterior Tentorial Stitch.

Additionally, we take an anterior tentorial stitch using 5-0 Prolene to retract the anterior tentorial leaflet. This helps to increase the surgical corridor and allows tumor visualization and removal from the undersurface of tentorium.

### 7:04 Dissection of Tumor From Cranial Nerve IV.

Using fine microdissectors the trochlear nerve is meticulously separated from the tumor.

### 7:31 Dissection of Tumor From Brainstem and Perforators.

Maintaining the arachnoid plane is of paramount importance in meningioma surgery. Using a combination of sharp as well as blunt dissection, the tumor is dissected slowly from the brainstem and its perforators.

### 7:45 Dissection of Tumor From Cranial Nerves VI, VII, and VIII.

Maintaining the arachnoid plane, the tumor is dissected from the seventh-eighth nerve complex as well as the sixth cranial nerve.

### 7:58 Preservation of Cranial Nerves.

This image depicts the fifth, sixth, seventh, and eighth cranial nerve preservation after meticulous tumor dissection. The fifth cranial nerve forms the center of the MDK anterior petrous rhomboid exposure, and the entire tumor, which was superior as well as inferior to the trigeminal nerve, was removed.

### 8:22 Identification of Basilar Trunk.

The basilar trunk in the prepontine cistern was identified.

### 8:33 Repair of Dural Defect.

The repair of the dural defect is important to prevent CSF leak in the postoperative period. The basal temporal dura is repaired primarily using 5-0 Prolene, and the posterior fossa dural defect is plugged using a combination of fat, fascia, and fibrin glue.

### 9:02 Immediate Postoperative Computed Tomography (CT) and Hospital Course.

This is the postoperative noncontrast CT scan of the patient. In the immediate postoperative period, the patient had transient sixth nerve paresis. The lower cranial nerve deficit was the same as in the preoperative period. No other cranial nerve deficit was seen. She had uneventful postoperative recovery.

### 9:20 Postoperative Imaging.

The postoperative contrast-enhanced MRI of the patient at 1-year follow-up shows no evidence of recurrent or residual lesion.

### 9:29 Comparison of Preoperative and Postoperative CT.

The MDK anterior petrous rhomboid approach remains a versatile approach for petroclival meningiomas which are not extending lateral and inferior to the IAC.^3^ Pre- and postoperative CT images highlight the operative corridor created by the drilling of the rhomboid.

### 9:45 Follow-Up Clinical Status.

At 3 years’ follow-up, the patient is doing well and the hoarseness of voice and swallowing difficulty have improved postsurgery.

## Acknowledgments

We thank Mr. Shashi Shekhar and Mr. Trivender Yadav for their assistance in editing this video and preparing the transcript.

## Disclosures

The authors report no conflict of interest concerning the materials or methods used in this study or the findings specified in this publication.

## Author Contributions

Primary surgeon: Suri. Assistant surgeon: Sharma, Raheja. Editing and drafting the video and abstract: Sharma, Katiyar, Raheja. Critically revising the work: Suri, Sharma, Raheja. Reviewed submitted version of the work: Suri, Sharma, Raheja. Approved the final version of the work on behalf of all authors: Suri. Supervision: Suri, Raheja.
